# Transmission sources and severe rat lung worm diseases in travelers: a scoping review

**DOI:** 10.1186/s40794-022-00184-4

**Published:** 2023-02-10

**Authors:** Atibordee Meesing, Sittichai Khamsai, Kittisak Sawanyawisuth, Somsak Tiamkao, Wanchai Maleewong, Panita Limpawattana, Bundit Sawunyavisuth, Chetta Ngamjarus, Watchara Boonsawat

**Affiliations:** 1 Department of Medicine, Faculty of Medicine, Khon Kaen University, 123 Mitraparp Road, Khon Kaen, Thailand 40002; 2 Department of Parasitology, Faculty of Medicine, Khon Kaen University, Khon Kaen, Thailand 40002; 3 Department of Marketing, Faculty of Business Administration and Accountancy, Khon Kaen University, Khon Kaen, Thailand 40002; 4 Department of Epidemiology and Biostatistics, Faculty of Public Health, Khon Kaen University, Khon Kaen, Thailand 40002

**Keywords:** African giant snails, Pila snails, Apple snails, *Angiostrongylus cantonensis*

## Abstract

**Background:**

Rat lung worm disease (RLWD) has several clinical forms including eosinophilic meningitis (EOM) and two severe forms, eosinophilic meningoencephalitis (EOME) and eosinophilic radiculomyelitis (EORM). It remains unclear whether transmission sources are associated with severe forms of RLWD. This study aimed to evaluate if transmission factors are related to the severity of RLWD among travelers by using a scoping review of case reports.

**Methods:**

This was a review using five databases to retrieve case reports and case series of travelers with RLWD. Clinical data and transmission sources of reported cases diagnosed as RLWD were retrieved. The outcome of the study was occurrence of severe forms of RLWD defined as EOME, EORM, and combined EOME/EORM.

**Results:**

We retrieved 1,326 articles from five databases and 31 articles were included in the analysis. There were 84 cases eligible from 15 countries. Four cases were excluded. Seventy cases were in EOM group and 10 cases had EOME or EORM. Compared with the EOM group, the EOME, EORM, and combination EOME/EORM group had similar age, sex, and risk factors of consumptions of apple snails, shrimp and prawn, and salad/vegetables. The EOME group had higher proportion of consumption of African snails than the EOM group (60% vs 13.8%). However, only one study reported the consumption of African snails and the heterogeneity between studies and the small sample size impeded direct comparisons between groups.

**Conclusions:**

RLWD in travelers can be found in most continents and mostly get infected from endemic countries of RLWD. Further studies are required to evaluate the association between transmission vectors and severity of RLWD.

**Supplementary Information:**

The online version contains supplementary material available at 10.1186/s40794-022-00184-4.

## Introduction

Rat lung worm disease (RLWD), caused by *Angiostrongylus cantonensis* infection, is a global neurological disease. There are three main forms of the RLWD, namely, eosinophilic meningitis (EOM), eosinophilic meningoencephalitis (EOME), and ocular angiostrongyliasis [1, 2]. In addition, there are other forms of RLWD in the literatures, for example, gastrointestinal involvement, eosinophilic radiculomyelitis (EORM), and otoangiostrongyliasis [3–5]. These neurological symptoms may be resolved or persistent causing morbidity or mortality [6]. A previous study in Thailand reported that the mortality rate of EOME, a severe form, was as high as 80% [7].

In addition to the people in the endemic areas [8], travelers are another group of persons at risk for RLWD [9]. It may be difficult for physicians to diagnose RLWD if history of traveling and exposure to *A. cantonensis* larvae is ignored [1, 6, 10, 11]. Humans can get RLWD by consumption of *A. cantonensis* containing foods including raw freshwater snails, fish, shrimp, or vegetables [12, 13]. Consumption of specific food (transmission sources) may cause severe RLWD. A previous study found that eating raw monitor lizard is associated with EOME in six patients as well as a case of eating raw frogs with wine [14–16]. However, there is no previous study on an association between transmission sources and severity of RLWD particularly in travelers. This scoping review aimed to describe transmission sources and severity of RLWD in travelers.

## Methods

This was a scoping review by using five databases including PubMed, Scopus, Central database, ProQuest, and CINAHL database [17]. We included a case report or case series of travelers with RLWD. Those articles with an observational design, meta-analysis, or intervention study were excluded. The full search details were in Additional file 1: Appendix. The last search was performed on April 1, 2022. Searching included any articles published since the beginning of the database through April 1, 2022. Hand search was also applied to find additional online articles or data. Eligible articles were included after duplication removal. An initial screening was conducted to include relevant articles. Full text review was performed in those relevant articles; those reports in non-travelers were excluded. The extraction processes were executed by two independent authors (AM and SK). The diagnosis of RLWD was done clinically or by confirmation tests such as ELISA or other serological tests.

Clinical data and risk factors of RLWD were collected. The patients whose clinical data were not available were excluded. The studied variables included baseline characteristics and the risk factors including age, sex, transmission sources for RLWD. The transmission sources defined as raw or uncooked freshwater snails, slugs, shrimp/prawn, frogs, crabs, fish, monitor lizards, vegetables, salad, or water/juice. Study characteristics on time to diagnosis or incubation period, time to treatment, diagnosis of RLWD, confirmation test, treatment, and treatment outcomes were studied. An outbreak investigation was also reported. The outcome of the study was the occurrence of RLWD which was categorized as EOME, EORM and the combination of the two (EOME/EORM). Severe consequences including death and coma were also retrieved. Factors associated with the occurrence of EOME, EORM, and combined EOME/EORM were analyzed and reported using descriptive statistics. The statistical analyses were performed by STATA software version 10.1 (College Station, Texas, USA).

## Results

**Fig. 1:**
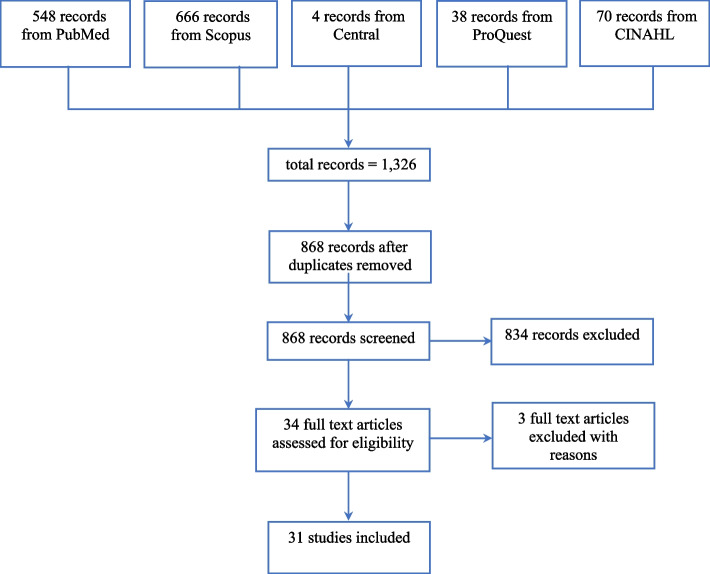
A prisma flow chart of reported cases of rat lung worm disease caused by *Angiostrongylus cantonensis* in travelers

There were 1,326 related articles from five databases and 868 articles for screening as shown in the Prisma flow (Fig. 1). Of those, 834 articles were excluded due to non-relevance. There were 34 articles for full text review with three articles excluded due to non-traveler studies. In total, there were 31 articles included in the review [4, 18–47] with 84 patients from 15 countries around the world (Table 1). Thailand, USA, and South Korea were the top three countries with 25, 17, and 16 patients respectively. There were eight countries from Europe, two countries from Asia (China and Singapore), one country from Australasia (Australia), and another one country from South America (Brazil). Cases were reported from 1982 until 2019. The mortality rate was 2.38% (2 patients) with severe consequences in 5 patients (5.95%).

**Table 1:** Country and numbers of reported cases of rat lung worm disease caused by *Angiostrongylus cantonensis* in travelers (*n* = 84)

Country	Year	Numbers (%)	Infected country	Suspected foods	Mortality/Severe consequences
Thailand	2001, 2011, 2018	25 (29.76)	Taiwan	Apple snails	1 coma
USA	1983, 2001, 2002, 2004, 2009, 2017	17 (20.24)	Jamaica, Tongo, Domincan Republic, South Pacific, USA^a^	Salad, raw snails, lettuce, crab, shrimp	1 severe
South Korea	1982	16 (19.05)	American Samoa	African snails	1 death
France	1988, 1996, 2002, 2008	8 (9.52)	French Polynesia	Prawn, raw fish	
Australia	1990, 1999	4 (4.76)	Fiji	Prawn	1 death
Switzerland	1995, 2004, 2019	3 (3.57)	French Polynesia, Cuba, Thailand	Raw fish	
Netherland	2015	2 (2.38)	Philippines	Salad, prawns	
Germany	2006, 2009	2 (2.38)	Dominican Republic, Thailand	Fish, clam, vegetables, salad	
Brazil	2004	1 (1.19)	Fiji	Vegetables	1 severe
Belgium	2007	1 (1.19)	Fiji	Salad, prawns	
Italy	2007	1 (1.19)	Dominican Republic	Shrimp	1 severe
UK	2007	1 (1.19)	Thailand	Snails, salad	
Croatia	2009	1 (1.19)	Malaysia	Vegetables, shrimp	1 severe
China	2019	1 (1.19)	China^a^	NA	
Singapore	2004	1 (1.19)	Korea	Salad	

*NA* Not available

^a^Different part of country

There were three major outbreaks reported with the highest numbers of patients [23, 27, 47]. The first outbreak was reported from Samoa by Kilks et al. in 1982 [47]. Sixteen out of 24 Korean fishermen (66.7%) who ate raw or uncooked snails developed RLWD: EOM or EORM. Those who ate cooked or did not eat snails did not have the RLWD. Three out of 16 (18.8%) had EOME: one patient died (33.3%).

Another outbreak from Taiwan was reported with 17 Thai patients [23]. The patients consumed raw apple snails from the ponds. All patients diagnosed as EOM and presented with acute headache, but only 11 patients had neck stiffness (64.7%) and 3 patients (17.6%) had pathognomonic hyperesthesia [11]. None of the patients developed severe EOME form. After treatment, all patients had an improvement from headache with minimal sequelae.

The final outbreak from the US reported 12 out of 23 medical students (52.2%) traveled to Jamaica [27]. All patients presented with headache and were diagnosed as EOM: nine patients were admitted (75.0%). Out of 12 patients, neck stiffness and hyperesthesia were found in 10 (83.3%) and 6 (50.0%) patients, respectively. Caesar salad was reported to be associated with the outbreak (*p* = 0.007). No consumption of snails or slugs was reported by the patients. Headache was improved with one patient reported of fine tremor of the arms and legs.

There three outbreak investigations to assess the possible causes of RLWD by Slom et al. in Chicago, Wang et al. in Taiwan, and Kliks et al. in Hawaii [27, 36, 47]. Slom et al. investigated on 23 travelers returning from Jamaica, twelve of them diagnosed with RLWD (cases) and eleven without infection (controls). The authors used a structured questionnaire on food consumed in Jamaica and linked eating in one specific restaurant and Caesar salad to RLWD (*p* = 0.001 and 0.007, respectively). Caesar salad contained romaine lettuce from the US and salted anchovies, no snails or mollusks were reported. The authors from Taiwan reported three patients consumed wine with snails from an irrigation canal. The snail meat was either seasoning or roasted for 5–10 min. The authors evaluated if there were any *A. cantonensis* larva in five irrigations. *A. cantonensis* larvae were found in snails of all five irrigations with the infected rate of 12.1%, 14.9%, 17.9%, 26.1%, and 29.4%. The larvas were still after 120 min of seasoning or 20 min of roasting. Finally, another study evaluated numbers of snails consumed in the infected patients. The numbers of raw snails eaten by 9 out of 12 patients were reported: ranged from 2 to 5 snails.

Regarding study characteristics on diagnosis and treatment (Table 2), the incubation period was between 1 and 80 days. Cerebrospinal fluid (CSF) eosinophils ranged between 0 and 73%, while CSF white blood cells were reported from 0 to 2,800 cells/mm^3^. Note that CSF eosinophilia was not detected in the first lumbar puncture but was presented in the later lumbar puncture [27, 41]. Most studies used serum antibodies against *A. cantonensis* to 31 kDa antigenic bands, only two studies had recovered *A. cantonensis* larva in either CSF or pulmonary arteries [22, 23]. Corticosteroid and repeated lumbar puncture was the treatment regimen for severe cases. The clinical outcomes were mostly favorable except those with severe diseases. The recovery time was about 4–8 weeks in average.

**Table 2:** Study characteristics regarding diagnosis and treatments of rat lung worm disease caused by *Angiostrongylus cantonensis* in travelers (*n* = 84)

Country (ref)	Time to diagnosis	Time to treatment	CSF white blood cells (cells/mm^3^)	CSF eosinophils (%)	Confirmation test (no positive/total patients)	Treatment	Outcomes
Taiwan [23]	4–23 days	NA	0–1660	0–20	Serum Ab (16/17) CSF Ab (5/17)^b^	Steroid	Recovered
Taiwan [24]	3–80 days	NA	0–1660	0–73	Serum Ab (30/31) CSF Ab (5/17)	Steroid	Recovered with 2 deaths
USA [18]	4 weeks	2 weeks	152	25	NA	Aspirin, acetaminophen	Improved 8 days
USA [25]	5 days	4 weeks	246	3	Serum Ab to 31 kDa	None	3 months
USA [27]	6–31 days	NA	18–765	0-54^a^	Serum Ab to 31 kDa (11/12)	Opioids, NSAIDs, steroid, LP	Improved 4–8 weeks
USA [30]	NA	3 days	278	61	Serum and CSF to 31 kDa	Brufen, gabapentin	Improved 8 weeks
USA [38]	1.5 months	1 month	270	12	CSF PCR	LP, steroid	Improved 2 months
USA [41]	5 weeks	2 weeks	NA	1^st^: 55 (0) 2^nd^: 754 (44)	Serum, CSF convalescent Ab to 31 kDa	Albendazole	NA
USA [47]	1–6 days	NA	12–2800	10–35	Serum ELISA (10/10)	Thiabendazole	Recovered 4–6 weeks with one death
France [35]	NA	14 days	1400	35	Serum convalescent Ab	Steroid, ivermectin	Improved 2 months with relapse at 6 months
Australia [22]	NA	6 days	55	0	Autopsy larva in pulmonary artery	IV methylprednisolone	Death
Australia [29]	NA	1 week	435	45	Serum Ab to 31 kDa	NA	Intubated
Switzerland [28]	Few days	NA	502	23	Serum Ab to 200, 95, 55, 31 kDa	NA	NA
Switzerland [44]	2 weeks	NA	1067	25	Serum convalescent Ab	Albendazole, steroid	Improved 2 weeks
Netherland [37]	NA	7 days	1323	0	CSF PCR	NA	NA
Germany [31]	1 weeks	3 weeks	NA	NA	CSF Ab	Oral albendazole, steroid	Improved 4 weeks
Croatia [4]	35 days	17 days	320	6.5	Immunoblotting 31 kDa	Repeated LP	Improved 1 month
Belgium [34]	NA	27 days	342	40	Serum Ab	IV methylprednisolone	Improved 5 months

*NA* Not available, *Ab* Antibodies, *CSF* Cerebrospinal fluid, *PCR* Polymerase chain reaction, *LP* Lumbar puncture

^a^Some patients had CSF eosinophils in the later LP

^b^Larva detected in the CSF

Among 84 patients, four patients were excluded due to the absence of clinical data in the report. Most patients had EOM (70 patients; 87.50%), while 7 patients had EOME and 4 patients had EORM. Note that one patient had both EOME and EORM (Table 3). Compared with the EOM group, the EOME, EORM, and combination of EOME/EORM group had comparable age, sex, or risk factors of consumptions of apple snails, shrimp and prawn, and salad/vegetables (Table 2). The EOME group had a higher proportion of consumption of African snails than the EOM group (60.00% vs 13.85%) but there were comparable proportions of consumptions of African snails in the EORM and combination of both conditions group versus EOM group (Table 3).

**Table 3:** Baseline characteristics and risk factors for eosinophilic meningoencephalitis (EOME), eosinophilic radiculomyelitis (EORM), or combination of eosinophilic meningoencephalitis and radiculomyelitis (EOME/EORM) caused by *Angiostrongylus cantonensis* in travelers

Factors	EOME		EORM		EOME/EORM	
	EOM *n* = 70	EOME *n* = 7	EOM *n* = 70	EORM *n* = 4	EOM *n* = 70	EOME/EORM^a^ *n* = 10
Median age (range), years	29 (1–55)	21 (1–40)	29 (1–55)	45 (1–47)	29 (1–55)	30 (1–47)
Advanced age > 60 years	1 (3.13)	2 (33.33)	1 (3.13)	1 (25.00)	1 (3.13)	2 (22.22)
Male	56 (80.00)	6 (85.71)	56 (80.00)	3 (75.00)	56 (80.00)	8 (80.00)
African snails	9 (13.85)	3 (60.00)	9 (13.85)	0	9 (13.85)	3 (37.50)
Apple snails	24 (36.92)	1 (20.00)	24 (36.92)	0	24 (36.92)	1 (12.50)
Shrimp and prawn	12 (17.65)	0	12 (17.65)	2 (66.67)	12 (17.65)	2 (25.00)
Salad and vegetables	19 (27.94)	1 (20.00)	19 (27.94)	1 (33.33)	19 (27.94)	2 (25.00)

Data presented as number (percentage) unless indicated otherwise

^a^One patient had both EOME and EORM

## Discussion

RLWD has been reported in travelers worldwide in almost all continents except in Africa. The most common countries of RLWD are Thailand, USA, and South Korea accounted for 69.0% (58/84 cases) as shown in Table 1. Not only reported from other countries, Thailand is the most endemic are for RLWD [48]. There were 1337 reported cases out of 2827 cases worldwide (47.3%), while French Polynesia and USA were ranked as the third and fourth endemic countries resulting in several reported cases in travelers infected from and to these two countries (Table 1). There is no reported case from Africa may be due to limited travel in this continent as well as no reported cases previously. Note that study characteristics in travelers were comparable with previously reported. The maximum incubation period was slightly shorter than the studies in Thailand which had incubation at the most of 3 months [10–12]. CSF eosinophils, a diagnostic indicator, may be found in the later lumbar punctures as well as the convalescent serum antibodies against the *A. cantonensis* antigenic bands.

This study revealed that people infected RLWD by African giant snails had higher rate of EOME than the EOM group. Despite consumption of less than 10 African snails per patient, three of 12 Korean fishermen (25%) developed severe EOME [47]. However, only one study reported the consumption of African snails and the heterogeneity between studies and the small sample size impeded direct comparisons between groups. (Table 2). Patients with RLWD in Thailand usually have a history of eating raw freshwater snails such as *Pila* snails with quite a few number of snails [49, 50]. Additionally, very few cases of severe or EOME were reported in Thailand. A review of 527 patients with EOM found that none of them had severe RLWD or EOME [49], while reports from outside Thailand had more severe consequences such as EOME associated with African snails [51–53]. Compared with other transmission sources, African giant snails accounted for 17.4% of all sources (12 out of 69 cases) as shown in Table 2. Note that only one case series reported on consumption of African giant snails in this review [47].

Presence of EORM is another factor for severe disease [47]. For EORM and combination of EOME/EORM outcomes, there is no significant factor associated with these outcomes. These findings may be due to small number of patients in EORM category (Table 2). The causative agent *A. cantonensis* is a neurotropic nematode but they usually migrate to meninges in humans. Random migration to spinal cord may cause severe disease as shown by one fatal case with the evidence of *A. cantonensis* larva in the lumbar spinal cord but not in the meninges or brain [47]. However, the number is too small to determine an association.

There are some limitations in this study. The design is a retrospective review of the database. Therefore, it may be some missing data shown in Table 2. Causal relationship may not be demonstrated. No correlation or meta-analysis was performed. Finally, in the EORM group had small sample size. Further larger studies are required.

## Conclusions

RLWD in travelers can be found in most continents and mostly get infected from endemic countries of RLWD. Further studies are required to demonstrate the association between transmission vectors and severity of RLWD.

## Supplementary Information

**Additional file 1: Appendix.** Searching strategies of reported cases of rat lung worm disease caused by *Angiostrongylus cantonensis* in travelers in five databases.

## Data Availability

Data are available upon request.
