# Supplementary material for: Transmission sources and severe rat lung worm diseases in travelers: a scoping review
Source: Trop Dis Travel Med Vaccines. 2023 Feb 10;9:2. doi: 10.1186/s40794-022-00184-4 (PMC9912548; doi:10.1186/s40794-022-00184-4)
Supplement: Supplementary file 1 — Additional file 1: Appendix. Searching strategies of reported cases of rat lung worm disease caused by Angiostrongylus cantonensis in travelers in five databases. [file 40794_2022_184_MOESM1_ESM.docx]

**Appendix: searching strategies of reported cases of rat lung worm disease caused by *Angiostrongylus cantonensis* in travelers in five databases.**

**Appendix 1: Searching strategy for PubMed on 1 April 2022**

| **Search number** | **Search terms** |
| --- | --- |
| 1 | "Angiostrongyliasis"[Title/Abstract] OR "Angiostrongylus cantonensis"[MeSH Terms] |
| 2 | "Humans"[Mesh] |
| 3 | #1 AND #2 |

**Appendix 2: Searching strategy for Scopus on 1 April 2022**

( TITLE-ABS-KEY ( angiostrongyliasis ) OR TITLE-ABS-KEY ( angiostrongylus AND cantonensis ) ) AND ( TITLE-ABS-KEY ( humans ) ) AND ( LIMIT-TO ( DOCTYPE , "ar" ) OR LIMIT-TO ( DOCTYPE , "re" ) ) AND ( LIMIT-TO ( EXACTKEYWORD , "Human" ) )

**Appendix 3: Searching strategy for Central on 1 April 2022**

| **Search number** | **Search terms** |
| --- | --- |
| 1 | MeSH descriptor: [Angiostrongylus cantonensis] explode all trees |
| 2 | (Angiostrongyliasis):ti,ab,kw |
| 3 | #1 OR #2 |

**Appendix 4: Searching strategy for ProQuest on 1 April 2022**

ti(Angiostrongylus cantonensis) OR ti(Angiostrongyliasis) OR ab(Angiostrongylus cantonensis) OR ab(Angiostrongyliasis) NOT animal* Additional limits - Document type: Article, Full text

**Appendix 5: Searching strategy for CINAHL 1 April 2022**

| **Search ID** | **Search Terms** | **Search Options** |
| --- | --- | --- |
| S1 | TI angiostrongyliasis OR AB angiostrongyliasis OR TI angiostrongylus cantonensis OR AB angiostrongylus cantonensis | Expanders - Apply equivalent subjects  Search modes - Find all my search terms |
